# Sustained exercise hyperemia during prolonged adenosine infusion in humans

**DOI:** 10.14814/phy2.14009

**Published:** 2019-02-25

**Authors:** Sushant M. Ranadive, John R. A. Shepherd, Timothy B. Curry, Frank A. Dinenno, Michael J. Joyner

**Affiliations:** ^1^ Department of Anesthesiology Mayo Clinic Rochester Minnesota; ^2^ Department of Kinesiology School of Public Health University of Maryland College Park Maryland; ^3^ Department of Health and Exercise Science and Center for Cardiovascular Research Colorado State University Fort Collins Colorodo

**Keywords:** Adenosine, blood flow, exercise, exercise hyperemia, vasodilation

## Abstract

The contribution of Adenosine (ADO) to exercise hyperemia remains controversial and it is unknown whether ADO can evoke the prolonged vasodilation seen during exercise bouts. Therefore, we tested hypotheses in the human forearm during 3 h of intra‐arterial high dose ADO infusion: (1) skeletal muscle blood flow would wane over time; (2) exercise hyperemic responses during ADO administration would be unaffected compared to baseline. Using sodium nitroprusside (SNP), we tested parallel hypotheses regarding nitric oxide (NO) in a separate group of participants. Seventeen young healthy participants (ADO:* n *=* *9; SNP:* n *=* *8) performed multiple rhythmic handgrip exercise bouts (20% of maximum), two during saline and five during 3 h of continuous drug infusion. Five minutes of ADO infusion resulted in a ~5‐fold increase in forearm vascular conductance (FVC; 4.8 ± 0.6 vs. 24.2 ± 3.2 mL/min/100 mmHg, *P *<* *0.05). SNP caused a ~4‐fold increase (4.4 ± 0.6 vs. 16.6 ± 2 mL/min/100 mmHg, *P *<* *0.05). FVC did not wane over time with ADO (24.2 ± 3.2 and 22 ± 1.2 mL/min/100 mmHg [*P *>* *0.05]) or SNP (16.6 ± 2 and 14.1 ± 2.4 mL/min^/^100 mmHg [*P *>* *0.05]) at 5 versus 150 min. Superimposed exercise during ADO or SNP infusions evoked marked and consistent additional dilation over the course of the infusions. Our findings demonstrate that in humans there is no reduction in endothelial or vascular smooth muscle responsiveness to the exogenous vasodilatory metabolites ADO and NO. Additionally, even in the presence of an exogenous vasodilator, superimposed exercise can cause significant hyperemia.

## Introduction

Skeletal muscle blood flow increases during exercise in order to match the metabolic demands of the exercising muscle (Shepherd 1983; Saltin et al. 1998; Joyner and Casey 2015). This increase in blood flow to the exercising muscle is likely evoked via numerous locally active vasodilating substances. Consequently, the role and contribution of individual vasodilators have been a topic of investigation and discussion for the past century. In a recent study, we showed that prolonged infusions of adenosine triphosphate (ATP), a vasoactive metabolite that may play a significant role in exercise hyperemia, can evoke marked and prolonged vasodilation in the human forearm (Shepherd et al. 2016). Of note, the hyperemic responses to exercise superimposed on ATP‐mediated dilation were unaffected (Shepherd et al. 2016). This suggests that under these experimental conditions, non‐ATP dilating mechanisms were likely engaged to cause exercise hyperemia.

In this context, adenosine (ADO) is one of the commonly studied and discussed vasodilators that might play an essential role in exercise hyperemia (Hester et al. 1982; Saltin et al. 1998; Joyner and Casey 2015). However, in recent years the role of ADO during exercise hyperemia has been debated due to the following observations: (1) blocking ADO deaminase does not increase the blood flow to exercising muscles (Martin et al. 2007), (2) there is a dichotomous response in humans to exogenous ADO infusion, with “responders” and “non‐responders” despite similar exercise hyperemia between the groups (Martin et al. 2006a), (3) even though ADO‐mediated vasodilation is significantly blunted with nitric oxide synthase (NOS) inhibitors in responders; the exercise hyperemia is not blunted during NOS inhibition in either responders or non‐responders (Martin et al. 2006a), and (4) data from our laboratory have shown that the response to exogenous ADO administration does not correlate to the hyperemic responses to exercise (Martin et al. 2007). However, these studies evaluated the role of ADO during shorter periods of vasodilation and exercise. In terms of prolonged vasodilation in response to exogenous ADO infusion, Hester et al. (1982) reported desensitization (tachyphylaxis) to the vasodilator effects of ADO when infused at ~1000 times the normal resting venous level in an isolated dog gracilis muscle preparation for 150 min. However, this desensitization to exogenous ADO had no effect on the blood flow and vasodilator responses to electrically induced muscle contraction, suggesting a limited of role of ADO in prolonged vasodilation (Hester et al. 1982). To our knowledge there are no data in humans regarding whether (1) prolonged ADO infusion results in tachyphylaxis and (2) superimposed exercise (during the exogenous ADO infusion) evokes a normal vasodilator response.

With this information as a background, the primary hypothesis of the present investigation was that in humans the vasodilation caused by a high dose of exogenous ADO infusion would wane over time. The secondary hypothesis was that the hyperemic response to exercise would be unaffected during exogenous ADO infusion. Additionally, we used a second dilating substance (sodium nitroprusside, SNP) as a NO donor to evaluate if results from the ADO experiments were metabolite infusion specific or due to elevated background blood flow.

## Methods

### Subjects

A total of 17 young healthy recreationally active subjects (ADO: *N *=* *9; 6 men/3 women; SNP: *N *=* *8; 4 men/4 women) volunteered to participate in the study after providing written informed consent. All participants were free of acute and chronic cardiovascular or respiratory disease, not taking any medication or supplements, and all non‐smoking and non‐obese (body mass index; BMI < 30 kg/m^2^). All participants refrained from exercise, alcohol and caffeine for at least 24 h and fasted for 12 h before the start of the study. To control the effects of the reproductive hormones on cardiovascular function, all female subjects were studied during the early follicular phase of the menstrual cycle or the placebo phase of oral contraceptives and all had a negative pregnancy test the morning of the study day. Women with an inter‐uterine device were excluded. All study procedures were approved by the Institutional Review Board of the Mayo Clinic and performed according to the Declaration of Helsinki.

### Experimental protocol

**Figure 1:**
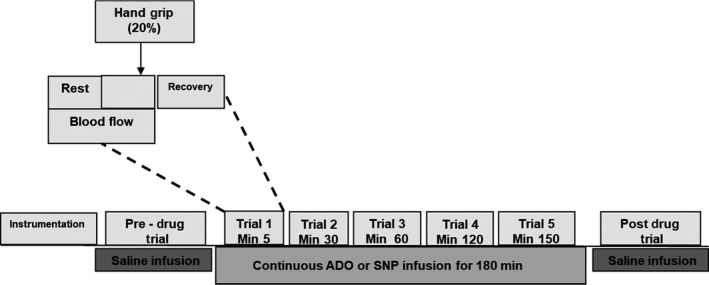
Overview of the experimental timeline. Schematic diagram showing overview of experimental timeline. ADO, adenosine; SNP, sodium nitroprusside.

Figure 1 shows the schematic of general experimental overview. The study was performed in the Clinical Research Unit (CRU) at the Mayo Clinic where ambient temperature was controlled between 22 and 24°C. On arrival to the laboratory, demographics, pregnancy test (females), forearm volume, and maximal voluntary contraction were obtained. The forearm volume was determined using water displacement technique (Chromy et al. 2015). All subjects then underwent placement of arterial catheter using aseptic technique followed by a 20‐min rest period.

### Subject monitoring

#### Brachial arterial catheterization and blood pressure and heart rate

In all subjects a 20‐gauge, 5‐cm (model RA‐04020; Arrow International, Reading, PA) catheter was placed into the brachial arterial of the exercising non‐dominant arm of the participant under aseptic conditions after local anesthesia (2% lidocaine). A 3‐way port system connector was attached to the catheter in series and transducer (model PX600F, Edwards Lifescience, Irvine, CA) permitting simultaneous measurement of beat‐by‐beat brachial arterial pressure and drug administration as previously described in detail (Dietz et al. 1994). Heart rate (HR) was recorded via a continuous 3‐lead electrocardiogram.

### Forearm exercise bouts

The forearm handgrip exercise was performed with the participant in supine position and arm positioned at the heart level (~0°) extending on the exercise table. Each bout consisted of 5 min of rhythmic forearm exercise with a handgrip dynamometer at a moderate intensity of exercise (20% of Maximal Voluntary Contraction). The weight was lifted 4–5 cm over a pulley at a duty cycle of 1 sec contraction and 2 sec relaxation using a metronome. Each bout was monitored by laboratory personnel to ensure proper timing of contractions. This level of exercise was selected based on our previous studies showing that it evokes stable blood flow responses over time and can be performed repeatedly without fatigue in healthy subjects. This exercise level also limits the changes in systemic hemodynamics and reflex activation of the sympathetic nervous system on exercise hyperemia (Richards et al. 2012; Casey et al. 2015).

### Drugs administered

#### Saline (control) bout

A saline infusion at the individualized determined rate (2.88 for ADO and 1.92 for SNP mL/min based on forearm volume) was started after the rest period. Two minutes of baseline values were recorded followed by 5 min of rhythmic handgrip exercise, and 2 min of recovery. Blood velocities and diameters were obtained continuously.

#### Drug (ADO and SNP) bouts

Following a 20‐min rest period after the completion of saline trail, pre‐infusion Doppler measurements were obtained to confirm that blood flow had returned to baseline. Either a continuous infusion of ADO or SNP was then started for a total of 180 min. Subjects were asked to perform five rhythmic handgrip exercise bouts identical to the saline bout at 5, 30, 60, 120, and 150 min into the ADO or SNP infusion. Five minutes after the end of the ADO or SNP infusion an additional control saline exercise bout was performed. For both ADO and SNP, the infusions were designed to evoke increases in forearm blood flow similar to those seen during moderate rhythmic forearm exercise (Casey et al. 2013).

#### Adenosine

The ADO solution was prepared by the Mayo Clinic Research Pharmacy and diluted in isotonic saline to a concentration of 100 μg/mL from a stock solution of 3000 μg/mL. The infusion flow rate was calculated as dose rate × forearm volume/100/ADO concentration. This formula accounts for each participant's individual forearm volume in order to maintain a constant dose rate of 23 μg 100 mL forearm vol/min intra‐arterially for continuous 180 min using a Harvard infusion syringe pump. For each participant the individualized infusion rate was used for saline control infusions.

#### Sodium nitroprusside

The SNP solution was prepared by the Mayo Clinic Research Pharmacy and diluted in isotonic saline to a concentration of 50 μg/mL from a stock solution concentration of 200 μg/mL. The infusion flow rate was calculated as dose rate × forearm volume/100/NTP concentration. This formula accounts for each participant's individual forearm volume in order to maintain a constant infusion rate of 5 μg·100 mL forearm vol/min intra‐arterially for a continuous 180 min using a Harvard infusion syringe pump. For each participant the individualized infusion rate was used for saline control infusions.

### Forearm blood flow

Brachial artery mean blood velocity (MBV) and brachial artery diameter were determined with a 12‐MHz linear‐array Doppler probe (model M12L, Vivid 7, General Electric, in Milwaukee, WI). Brachial artery blood velocity was measured throughout each condition with a probe insonation angle previously calibrated to 60°. Brachial artery diameter measurements were obtained at end diastole between contractions during steady‐state conditions. This method has been previously used for several other studies in our laboratory (Ranadive et al. 2014; Casey et al. 2015). Forearm blood flow (FBF) was calculated as FBF = MBV × *π* × (brachial artery diameter/2)^2^ × 60 with MBV (cm/s) and brachial artery cross‐sectional area (cm^2^) and expressed as milliliters per minute (mL/min).

#### Forearm vascular conductance

FVC (forearm vascular conductance) was calculated as (FBF/mean arterial pressure) × 100 and expressed as mL/min/100 mmHg so that FVC will be quantitatively similar to the standard units of FBF.

### Data analysis

Data were collected at 250 Hz, stored on a computer, and analyzed off‐line with signal processing software (WinDaq; DATAq Instruments, Akron, OH and Powerlab; ADInstruments, Sydney, Australia). HR and mean arterial pressure (MAP) were analyzed from the electrocardiogram and the brachial artery pressure waveform, respectively. MBV, HR and MAP were determined by averaging the last 60 sec of each baseline and exercise bout during control and continuous vasodilator infusion. Arterial diameters were measured at baseline and in the last 15 sec of the exercise bouts. The change in FBF and FVC within a condition (saline, ADO, or SNP) was calculated as exercise FBF – baseline FBF and exercise FVC – baseline FVC at each of the exercise bouts.

Two‐way repeated measures of analysis of variance (ANOVA) were performed to test the significance between and within bouts. Following a significant *F* test, pair‐wise differences were identified using Tukey's post hoc test procedure. The significant level was set at *P* < 0.05 and all data values are presented as mean ± SEM unless stated otherwise.

## Results

All 17 participants completed the protocol. The mean demographics for the participants who completed the ADO trial was 29 ± 1 years, body weight 83 ± 5 kg, height 180 ± 3 cm, and BMI 25 ± 1 kg/m^2^. Mean forearm volume was 1052 ± 76 (range = 710–1360 mL). The mean demographics for the participants who completed the SNP trial was 29 ± 1 years, body weight 80 ± 5 kg, height 178 ± 3 cm, and BMI 25 ± 1 kg/m^2^. Mean forearm volume was 1061 ± 74 (range = 730–1330 mL).

### Systemic hemodynamic variables

The group data for systemic hemodynamic variables for baseline are presented in Table 1. Additionally, there were no significant systemic hemodynamic differences in MAP or HR between the ADO, SNP and during the saline control and exercise bouts (data not presented).

**Table 1:** Participant characteristics

Variable	ADO (*n *=* *9)	SNP (*n *=* *8)
Systolic blood pressure (mmHg)	115 ± 2	114 ± 3
Diastolic blood pressure (mmHg)	68 ± 2	71 ± 2
HR (BPM)	61 ± 3	60 ± 2
20% maximum value contraction (lbs)	21 ± 2	20 ± 2
Forearm volume (mL)	1052 ± 76	1040 ± 74
Infusion flow rate (mL/min)	2.43 ± 0.2	2.0 ± 0.2
Volume delivered (mL)	437 ± 32	374 ± 27

Values are means ± SE for all subjects. BPM, beats per minute; ADO, Adenosine; HR, heart rate; SNP, sodium nitroprusside.

### Effect of ADO or SNP infusion on resting forearm blood flow and FVC

**Figure 2:**
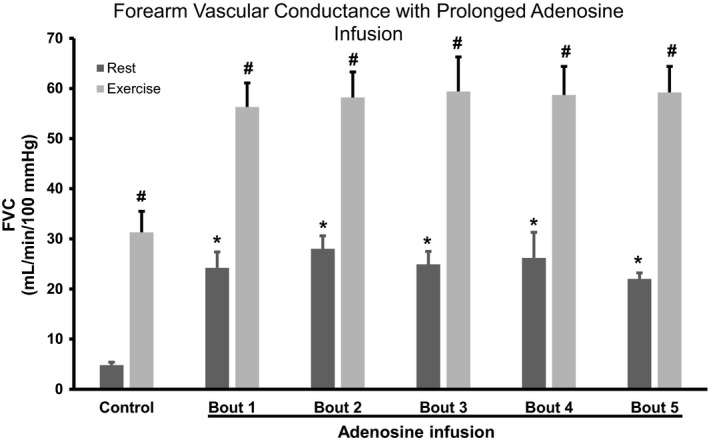
FVC with prolonged adenosine infusion. Values are expressed as means ± SE. *P <* *0.05 from control rest; #*P *<* *0.05 from baseline. FVC, forearm vascular conductance

**Figure 3:**
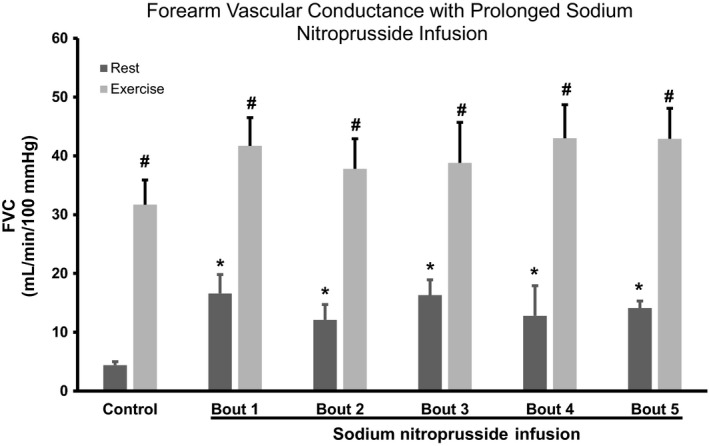
FVC with prolonged sodium nitroprusside infusion. Values are expressed as means ± SE. *P <* *0.05 from control rest; #*P *<* *0.05 from baseline. FVC, forearm vascular conductance.

The group data for FBF and the change in FBF and FVC from baseline are presented in Tables 2 and 3, whereas absolute FVC is presented in Figures 2 and 3. Five minutes of ADO infusion resulted in a significant increase in FVC (4.8 ± 0.2–24.2 ± 3.2 mL/min/100 mmHg/100 mL FAV, *P *<* *0.05) while FVC during SNP increased from 4.4 ± 0.6–16.6 ± 2.0 mL/min/100 mmHg/100 mL FAV (*P *<* *0.05). FVC responses during prolonged ADO or SNP infusion did not wane from minute 5 to minute 150 (24.2 ± 3.2 vs. 22.0 ± 1.2 mL/min/100 mmHg/100 mL FAV, *P *>* *0.05) and 16.6 ± 2 versus 14.1 ± 2.4 mL/min/100 mmHg/100 mL FAV, *P *<* *0.05, respectively.

**Table 2:** Adenosine: rest and exercise vascular hemodynamics

Variable	Saline (control)	Bout 1	Bout 2	Bout 3	Bout 4	Bout 5
Time (minutes)	‐	5	30	60	120	150
HR						
Rest	59 ± 2	60 ± 2	58 ± 2	61 ± 2	59 ± 2	59 ± 2
Exercise	63 ± 2	62 ± 2	63 ± 2	62 ± 2	64 ± 2	65 ± 2
MAP						
Rest	93 ± 3	92 ± 3	95 ± 3	97 ± 3	96 ± 3	96 ± 3
Exercise	98 ± 4	95 ± 3	96 ± 3	97 ± 4	102 ± 4	101 ± 4
FBF (mL/min/100 mL FAV)						
Rest	4.3 ± 0.6	20.9 ± 2.5*	24.8 ± 2.5*	22.4 ± 2.2*	25.8 ± 5.1*	20.4 ± 1.4*
Exercise	28.6 ± 4.3	52.0 ± 4.9*	54.2 ± 5.4*	55.5 ± 6.9*	55.7 ± 6.3*	56.3 ± 5.3*
(Δ) FBF from rest (mL/min/100 mL FAV)						
Exercise	24.3 ± 3.8	31.1 ± 5.7	29.4 ± 4.9	33.2 ± 6.3	29.9 ± 4.5	35.9 ± 5.0
Δ FVC from rest (mL/min/100 mmHg/100 mL FAV)						
Exercise	26.4 ± 3.7	32.1 ± 5.5	30.3 ± 4.7	34.5 ± 6.2	32.5 ± 4.3	37.2 ± 5.1

Values are means ± SE for all nine subjects. MAP, mean arterial pressure.

* *P *<* *0.05 from control. FBF, forearm blood flow; Delta FBF was calculated as bout exercise FBF – bout baseline FBF; FVC, forearm vascular conductance; Delta FVC was calculated as bout exercise FVC‐bout baseline FVC. 100.

**Table 3:** Sodium nitroprusside: rest and exercise vascular hemodynamics

Variable	Saline (control)	Bout 1	Bout 2	Bout 3	Bout 4	Bout 5
Time (minutes)	‐	5	30	60	120	150
HR						
Rest	55 ± 2	57 ± 2	57 ± 3	56 ± 2	56 ± 2	57 ± 2
Exercise	60 ± 2	63 ± 2	63 ± 3	63 ± 3	63 ± 2	64 ± 2
MAP						
Rest	90 ± 1	87 ± 2	91 ± 2	91 ± 2	93 ± 2	94 ± 2
Exercise	94 ± 1	93 ± 2	93 ± 1	94 ± 2	97 ± 2	98 ± 2
FBF (mL/min/100 mL FAV)						
Rest	4.0 ± 0.6	14.4 ± 1.6*	10.8 ± 1.8*	14.5 ± 1.9*	11.8 ± 1.8*	13.4 ± 2.0*
Exercise	29.4 ± 2.9	39.0 ± 2.5*	35.0 ± 2.6*	35.9 ± 2.8*	41.4 ± 2.8*	41.1 ± 3.0*
(Δ) FBF from rest (mL/min/100 mL FAV)						
Exercise	25.4 ± 3.1	24.6 ± 3.1	24.2 ± 3.7	21.4 ± 4.1	29.6 ± 3.1	27.7 ± 3.0
Δ FVC from rest (mL/min/100 mmHg)						
Exercise	27.3 ± 3.4	25.1 ± 3.3	25.6 ± 4.0	22.5 ± 4.2	30.2 ± 2.9	28.6 ± 3.3

Values are means ± SE for all eight subjects. HR, heart rate; MAP, mean arterial pressure.

* *P* < 0.05 from control. FBF, forearm blood flow; Delta FBF was calculated as bout exercise FBF – bout baseline FBF; FVC, forearm vascular conductance; Delta FVC was calculated as bout exercise FVC‐bout baseline FVC.

### Effect of exercise during ADO or SNP infusions on forearm blood flow and FVC

The group data for FBF and the change in FBF and FVC from baseline are presented in Tables 2 and 3, whereas absolute FVC is presented in Figures 2 and 3. FVC during exercise under control (saline) increased from 4.8 ± 0.6–31.3 ± 4.2 and further increased during ADO infusion from 24.2 ± 3.2–56.3 ± 4.8 mL/min/100 mmHg/100 mL FAV at bout 1 (*P *<* *0.05). Similar results were obtained with SNP. For example, FVC during exercise under control (saline) for the SNP trial increased from 4.4 ± 0.6–31.7 ± 3.2 mL/min/100 mmHg/100 mL FAV, *P *<* *0.05, and further increased during SNP infusion from 16.6 ± 2–41.7 ± 2.6 mL/min/100 mmHg/100 mL FAV at bout 1. Importantly, the magnitude of FVC response (resting to 5 min of exercise) was not significantly different between saline, ADO and SNP infusion (Tables 2 and 3).

The FVC responses evoked by exercise superimposed during the ADO infusion did not wane significantly over time with equivalent values between bout 1 of exercise versus bout 5 (56.3 ± 4.8 vs. 59.2 ± 5.2 mL/min/100 mmHg/100 mL FAV, *P *>* *0.05, respectively). Similarly, FVC responses evoked by exercise superimposed during the SNP infusion did not wane significantly over time during exercise with equivalent values between bout 1 of exercise versus bout 5 (41.7 ± 2.6 vs. 42.7 ± 3.0 mL/min/100 mmHg/100 mL FAV, *P *>* *0.05, respectively).

## Discussion

The primary findings of the present study are (1) exogenous infusion of ADO or SNP evoked robust forearm vasodilatory responses which did not wane over 180 min, (2) the magnitude of vasodilator response to exercise superimposed during the exogenous ADO or SNP infusion was similar as compared to saline, and (3) the vasodilator response to exercise did not wane over 180 min of ADO or SNP infusion.

To our knowledge, this is the first study to show that prolonged ADO infusion for 180 min in humans can evoke a consistent vasodilatory response, with no evidence of tachyphylaxis. Although we have previously demonstrated a potential dichotomy in the vasodilatory response between individuals to acute (minutes) and increasing doses of ADO (responders vs. non‐responders) (Martin et al. 2006b; Casey et al. 2009; Casey and Joyner 2011; Lopez et al. 2012), in the present study we observed prolonged and consistent vasodilation during exogenous ADO without any dichotomous results between participants. Additionally, the vasodilation during prolonged infusion of the NO donor compound, SNP, was also consistent over 180 min with no evidence of tachyphylaxis.

The model of 3 h infusion allowed us to test whether the blood flow and vasodilator response to ADO and SNP in humans would wane over 180 min. In isolated dog gracilis muscle, 3 h of exogenous ADO administration resulted in desensitization of the skeletal muscle blood vessels to ADO. Despite this observation, there was no effect on the vasodilator responses to electrically induced muscle contractions (Hester et al. 1982). Thus, in contrast to these findings by Hester et al., the data in present study suggest that in humans, ADO is capable of sustained vasodilation over 3 h. Therefore, in humans the ADO receptors may not undergo desensitization even when ADO is present in high concentration compared to resting conditions.

In addition to the prolonged and sustained vasodilation to both ADO and SNP, we found that the magnitude of the hyperemic and vasodilatory responses to exercise superimposed on the drug infusions were similar to that of exercise during saline infusion. One of the primary reasons for the similar vasodilation during exercise could be due to other dilating substances involved in the complex regulation of vascular tone during muscle contractions. In this context, K^+^‐mediated hyperpolarization in conjunction with local nitric oxide and prostaglandin release appears to be an essential component of the rapid dilator response seen at the onset of exercise and also contributes to the dilator responses seen during 5‐min bouts of exercise (Crecelius et al. 2013, 2014, 2015). It is likely that this mechanism of dilation is unaffected by the infusions of ADO and SNP. Along these lines, in a novel study, Terwoord et al. infused potassium chloride or SNP during rhythmic handgrip exercise. The authors reported potassium chloride infusion attenuated the vasodilatory response to exercise by ~30% whereas SNP infusion had no effect on the vasodilatory response of the subsequent exercise. Further, previous data from our laboratory have shown that blocking NOS in ADO responders results in significant blunting of ADO‐mediated vasodilation only at baseline, with no effect on exercise hyperemia (Martin et al. 2007). Taken together, these data suggest the idea that other vasodilatory mechanisms especially potassium in local vasodilation during exercise in humans is plausible (Mortensen et al. 2009; Terwoord et al. 2018).

It is also possible that the mechanical effects of contraction on blood flow were amplified when the vessels were vasodilated. Given that only modest increases in flow are generated via these effects under control conditions, it seems unlikely that they would be increased 5‐ to 10‐fold by the infusion of a vasodilator (Tschakovsky et al. 2002; Tschakovsky and Sheriff 2004; Kirby et al. 2007). Therefore, we favor the general explanation that combination of vasodilator mechanisms is responsible for the dilation seen when exercise was superimposed on top of the drug infusion. This may also support the theory that multiple vasodilators can increase or decrease responses if one is fully sustained or blocked.

### Experimental considerations

There are two limitations to our study. First, we did not perform a full saline control for 180 min rather only before and after drug administration. However, previous studies have shown that the dilator responses to repeated bouts of exercise are consistent over time (Casey et al. 2015). Second, it is possible that muscle pump could have partially contributed to the initial exercise hyperemia. As the baseline flow increased with infusion of ADO and SNP, with initiation of exercise there could have been suction or tethering of the blood vessels causing increased flow (Joyner and Casey 2015). However, it is important to note that the magnitude of change with exercise hyperemia was similar between saline and the vasodilator drugs. This finding is line with previous studies performed in a dog model with K^+^ infusion (Hamann et al. 2004). The authors had reported that muscle pump may not be the reason for contraction induced rapid hyperemia (Hamann et al. 2004). Therefore, the role of non‐metabolite component during superimposed exercise hyperemia even if present might be low and consistent between control and drug (Joyner and Casey 2015).

### Conclusions

Our findings demonstrate that in humans there is no reduction in endothelial or vascular smooth muscle cell responsiveness to the exogenous vasodilator metabolites ADO and NO. Additionally, even in the presence of exogenous vasodilator, superimposed exercise can cause significant exercise hyperemia.

## Conflict of Interest

No conflicts of interest, financial or otherwise, are declared by the author(s).
